# Supplementary material for: The COVID-19 Pandemic Sparked Off a Large-Scale Outbreak of Carbapenem-Resistant Acinetobacter baumannii from the Endemic Strains at an Italian Hospital
Source: Microbiol Spectr. 2023 Mar 23;11(2):e04505-22. doi: 10.1128/spectrum.04505-22 (PMC10101080; doi:10.1128/spectrum.04505-22)
Supplement: Supplemental file 5 — Fig. S1 and S2. Download spectrum.04505-22-s0005.pdf, PDF file, 1.0 MB [file spectrum.04505-22-s0005.pdf]

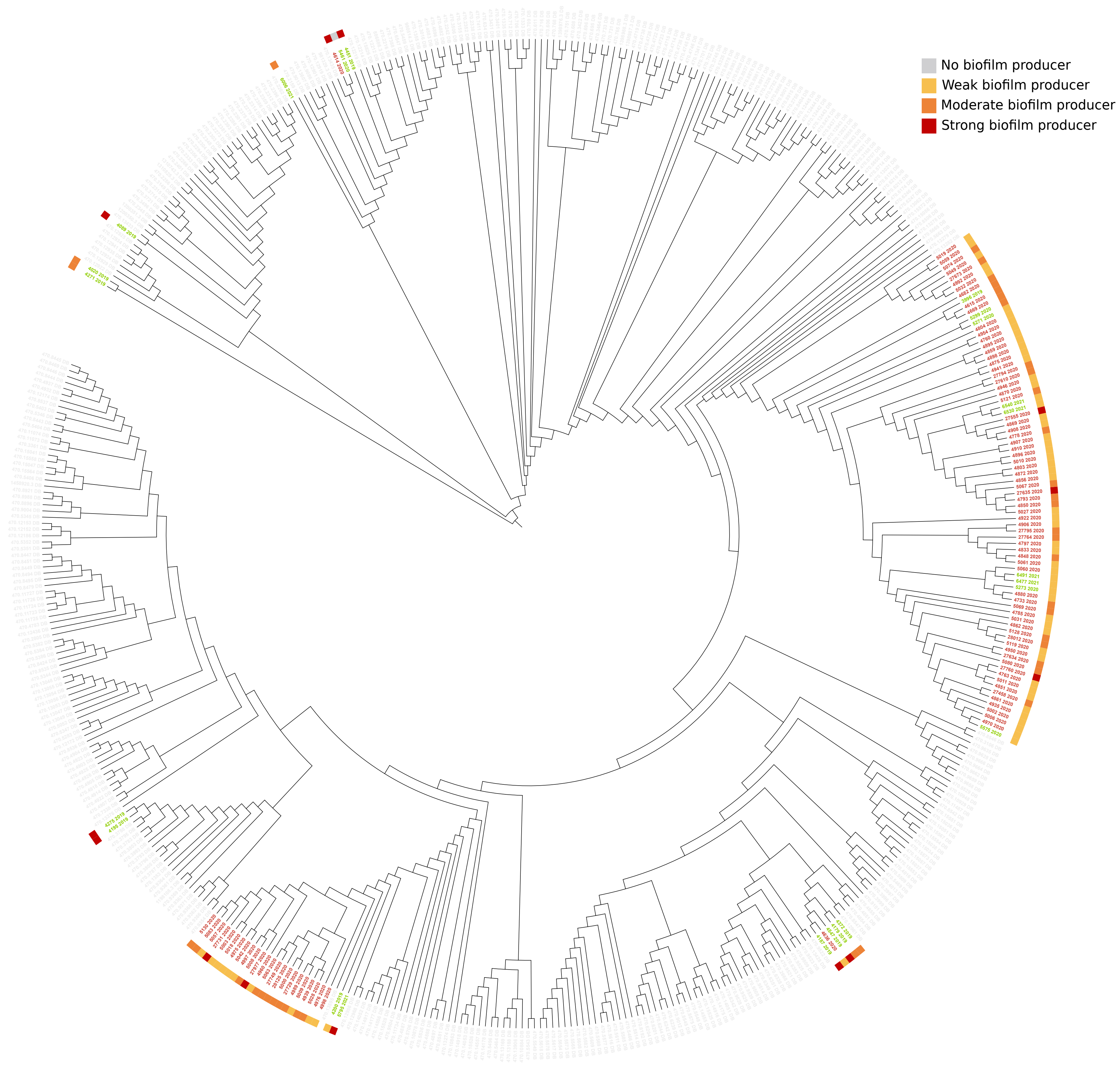


**Fig. S1** - Maximum likelihood phylogeny of 485 *A. baumannii* strains: 95 outbreak genomes (red labels), 23 surveillance genomes (green labels) and 367 PATRIC genomes (gray labels) inferred on coreSNPs with RAxML. The crown shows the biofilm production levels of each strain: no producers (gray), weak producers (yellow), moderate producers (orange), strong producers (red). This figure was obtained using iTol [https://itol.embl.de/].

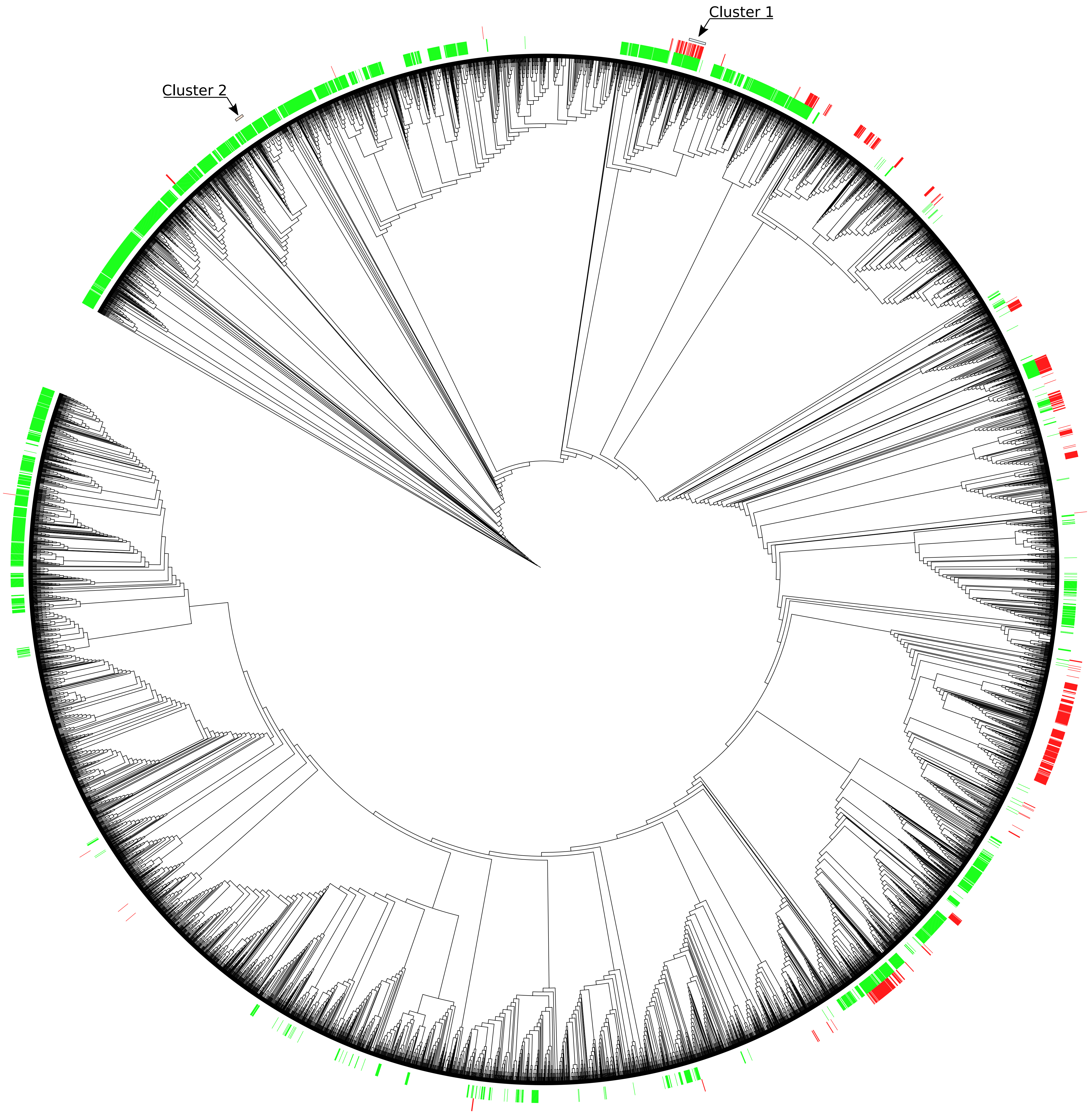


**Fig. S2** - Global phylogeny of 8969 strains of *A. baumannii*. The presence of 8 kb plasmid (green labels) and 100 kb plasmid (red labels) were determined by genomic analyses. This figure was obtained using iTol [https://itol.embl.de/].
